# Whole-Field Reinforcement Learning: A Fully Autonomous Aerial Scouting Method for Precision Agriculture

**DOI:** 10.3390/s20226585

**Published:** 2020-11-18

**Authors:** Zichen Zhang, Jayson Boubin, Christopher Stewart, Sami Khanal

**Affiliations:** 1 Department of Computer Science and Engineering, The Ohio State University, Columbus, OH 43210, USA; boubin.2@osu.edu (J.B.); cstewart@cse.ohio-state.edu (C.S.); 2 Department of Food, Agricultural and Biological Engineering, The Ohio State University, Columbus, OH 43210, USA; khanal.3@osu.edu

**Keywords:** convolutional neural networks, reinforcement learning, unmanned aerial systems, autonomous systems, precision agriculture, crop scouting

## Abstract

Unmanned aerial systems (UAS) are increasingly used in precision agriculture to collect crop health related data. UAS can capture data more often and more cost-effectively than sending human scouts into the field. However, in large crop fields, flight time, and hence data collection, is limited by battery life. In a conventional UAS approach, human operators are required to exchange depleted batteries many times, which can be costly and time consuming. In this study, we developed a novel, fully autonomous aerial scouting approach that preserves battery life by sampling sections of a field for sensing and predicting crop health for the whole field. Our approach uses reinforcement learning (RL) and convolutional neural networks (CNN) to accurately and autonomously sample the field. To develop and test the approach, we ran flight simulations on an aerial image dataset collected from an 80-acre corn field. The excess green vegetation Index was used as a proxy for crop health condition. Compared to the conventional UAS scouting approach, the proposed scouting approach sampled 40% of the field, predicted crop health with 89.8% accuracy, reduced labor cost by 4.8× and increased agricultural profits by 1.36×.

## 1. Introduction

It is predicted that the global population will increase to 9.7 billion in 2050 [1,2] and that agricultural production must double to meet the needs of this growing population and shift in dietary preference while balancing against energy and water constraints [3,4]. This goal cannot be reached by simply doubling the agricultural inputs because of constrained resources, already developed agricultural land limits and environmental concerns [5]. The future efficiency gains of agricultural production systems must be improved and adaptable to be able to increase yields with respect to large variances expected in weather locally and growing globally.

Precision agriculture (i.e., site-specific management practices) is a promising step towards improving efficiency and reducing adverse impacts of agricultural production [6]. It focuses on assessing variation across and within crop fields to divide a field into multiple management zones and treat each management zone accordingly [7,8]. Thus, it is critical to have spatial and temporal maps of crop and soil health in a timely fashion [9]. Accurate crop and soil health maps are critical to support site-specific management practices in a cost-effective manner. Management decisions based on inaccurate crop and soil health maps can result in unwanted crop yield loss, excessive fertilizer application and increased nutrient loads to waterbodies [10]. For instance, let us assume that a farmer applies fertilizers to only those crops that fall within the unhealthy zones—based on crop health maps. If crop health maps inaccurately label unhealthy areas as healthy, those misrepresented unhealthy sections of a field would not receive treatment, and thus crop yield of those areas could be poor. Alternatively, if healthy zones are mislabeled as unhealthy, they would receive unwanted fertilizer application, which means loss of farm resources as well as increased risk of nutrient runoffs and leaching without much increase in crop yield.

Accurate representation of field conditions via maps depends on temporal and spatial resolutions of data, which vary across sensors and platforms (e.g., satellite, weather stations and aircraft) used for data collection, which in turn influence data collection and processing costs [11]. Unmanned aerial systems (UAS) have emerged as a cost-effective approach for aerial scouting [12]. Compared to satellites, UAS can fly to waypoints, hover and collect high resolution data (millimeters per pixel) from large areas quickly. Compared to human piloted aircraft, UAS are 3× less expensive, achieve better spatial resolution and pose fewer safety risks [11]. Traditional UAS approaches for scouting involve a flying a grid-based flight pattern, capturing images comprising the entire crop field [12,13,14]. To scout a whole field, a UAS is given a set of waypoints (i.e., GPS coordinates) to follow, taking one picture at each waypoint. Various vegetation indices, such as excess green vegetation index (ExG) [15], are computed offline using visible images to indicate crop health conditions for each zone [16].

To provide accurate crop health information for a field, traditional exhaustive scouting approaches involve redundant data collection (i.e., 65–80% front and side overlap between images). Batteries on commodity UAS allow just 15–25 min of flight. UAS must land and recharge repeatedly to cover large fields. Human operators must monitor flights and battery capacity, swap and recharge batteries and possibly fly aircraft manually by remote control. These activities also delay missions. It can take a full 8-hours workday to exhaustively collect high definition images from every zone in an 80-acre crop field [11,17]. Thus, for UAS with onboard IoT systems, it is crucial to collect as much information as possible within a given UAS flight.

Autonomous systems sense and potentially alter their environment without human intervention. They manage IoT actuators (e.g., UAS flight controls) and machine learning to sense and identify phenomena in an effort to achieve high utility (e.g., low prediction error) outputs. Fully or partially autonomous tractors, planters and monitoring equipment already perform complex tasks in critical settings today. While autonomy can reduce labor costs and improve task performance, it also loses the robust problem-solving abilities of human operators, incurs engineering costs and is difficult to model (closed-loop systems). For example, Lin et al. relied on narrowly defined tasks to trace and model compute needs for autonomous cars [18]. Boubin et al. broadened Lin’s compute modeling by capturing environmental factors for UAS [17]. In-situ AI [19] and Boroujerdian et al. [13] generalized these approaches via environmental simulation.

Recent works in this space have focused on using machine learning to gather insights from UAS-collected images for a number of agricultural phenomena [20]. For example, Hunt and Rondon used NDVI thresholding and object based image analysis on UAS images to predict potato beetle damage [21]. Similarly, Alexandridis et al. used multi-spectral data and multiple image processing techniques to identify weeds (*Silybum marianum)* [22]. Han et al. used similar machine learning techniques to detect above ground biomass in corn [23]. These including other prior works in UAS remote sensing have relied on orthomosaic images, geometrically accurate composites of many images that depend on many UAS missions to construct as well as time consuming post-processing of images. Real-time remote sensing has also been performed in recent works. Yang et al. (2020) used a CNN and an adaptive, multi-height crop scouting algorithm to scout rice lodging in real time [24]. Besides, Boubin et al. (2019) [11] demonstrated early work in informed UAS sampling for crop yield modeling. Outside of agriculture, Zhang et al. used UAS and a single-shot detector for real-time vehicle detection [25]. Wang et al. adapted a deep reinforcement learning approach for autonomous UAS navigation in outdoor environment [26]. However, real-time map creation using UAS sampling in agriculture has not been deeply explored.

In this project, we show that, with the help of reinforcement learning (RL) and spatial ensembles of CNN [27,28], we can create accurate crop health maps and reduce flight time and costs by scouting only a fraction of a field. Specifically, we design a whole-field based fully autonomous aerial scouting system for UAS, an alternative to exhaustive scouting. In our approach, the UAS: (1) are piloted by software we designed; (2) can generate an accurate crop health map with only partial coverage of the field; and (3) can autonomously set their flight paths to maximize the accuracy of the crop health map. The latter feature distinguishes our approach from random sampling. In this work, we attempt to answer the following questions: (1) Can UAS autonomously select and fly over 20–40% of a field’s management zones and create accurate crop health maps? (2) By deploying whole-field RL algorithm, how much revenue can be gained from such cost savings in data collection?

## 2. Methods

### 2.1. Design

To reduce data collection and computation costs, we present a new RL approach, whole-field RL, to guide UAS in aerial crop scouting and compare the differences in crop health maps generated by these approaches with traditional methods as well as our previous work, local-field RL [11]. Since the traditional approach involves exhaustive scouting of a field, it is assumed that the crop health information based on this approach is 100% accurate, and thus used as ground truth data to evaluate findings based on two RL approaches.

**Figure 1:**
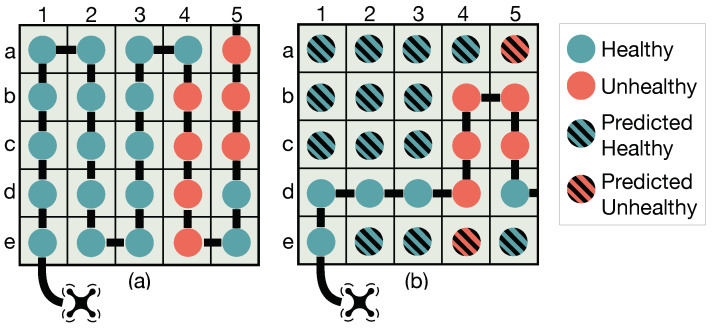
(**a**) Exhaustive scouting of a field wherein UAS visit all zones in a grid; and (**b**) fully autonomous aerial scouting wherein UAS visit a fraction of a field (i.e., eight zones) and predict crop conditions for unvisited areas.

Whole-field RL uses a full history of images captured by a UAS during a scouting mission and implements complex CNN models and a RL algorithm to extrapolate a whole-field crop health map from sensed data. That is, during a flight mission, whole-field RL uses all images (one image per zone) of previous UAS-visited zones as inputs to CNN models to construct crop health prediction maps which serve as inputs to the RL algorithm to decide the next zone to fly over. While effective, this is a computationally intensive approach (discussed in detail in Section 2.2). In contrast, local-field RL only uses one image from the last UAS-visited zone to predict its next path. After sampling enough points, whole-field RL then extrapolates crop health information for a whole field, returning a final map to the user. Compared to the traditional approach, which uses a predefined path for exhaustive data collection, both RL based scouting approaches visit less areas within a field and thus reduce data collection costs. For example, as shown in Table 1 and Figure 1, since UAS batteries drain at the same rate, exhaustive scouting incurs the cost of landing, recharging and flying back to its most recent zone. Exhaustive scouting takes two flights to generate the crop health map while fully autonomous aerial scouting only takes one flight. However, these approaches can introduce error as health conditions of the zones that are not directly observed are predicted. Thus, our work also focuses on minimizing prediction errors as well as finding a balance between prediction error and UAS coverage rate.

**Figure 2:**
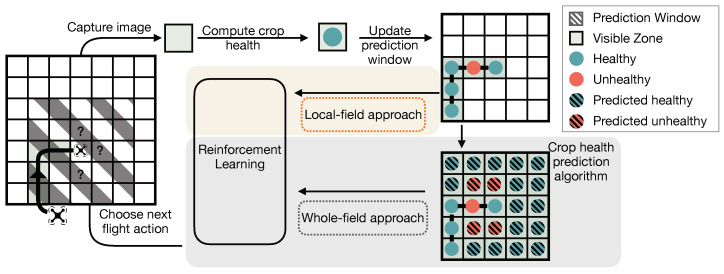
Difference between whole-field and local-field RL.

Figure 2 outlines our fully autonomous aerial scouting approach. First, UAS fly over management zones and collect images. Crop health is computed for each visited zone. We maintain a dataset of prior observations of crop health data. All visited zones, associated flight actions and their outcomes are stored as training data. Our RL algorithm computes the next flight action, wherein prior action and observation pairs predict the future utility of each candidate action, selecting the maximum. Utility is defined as the usefulness of an observation in accomplishing the current autonomy goal, as specified by a domain specific utility function. Here, utility gained after taking an action is defined as the improvement in crop health map accuracy. We use an unsupervised learning approach from prior work [17] based on k-nearest neighbors to determine the most similar prior observations from our dataset. We then compute the normalized utility gain garnered from taking each available flight action in our K similar observations. Utility gain, in this context, reflects the improvement in utility between two samples. Our RL approach seeks to maximize utility gain. Given extant crop health data, our RL algorithm computes mean utility gain for each candidate flight action using similar prior observations and chooses the action with the highest utility gain. For autonomous aerial scouting, the utility function seeks to maximize accuracy of the final extrapolated crop health map.

### 2.2. The Whole-Field RL Algorithm

The whole-field RL approach has three components: (1) a CNN to model crop health; (2) an algorithm to extrapolate crop health predictions over a whole field; and (3) a RL approach to improve future outputs. As an overview, CNN used spatial information of ExG to improve crop health prediction accuracy in areas scouted by UAS. The algorithm then expands predictions beyond spatial neighbors. Then, RL chooses flight paths that wisely sample zones to maximize the accuracy of predicted crop health maps (details provided later in this section). Finally, when desired coverage is reached, CNN-based crop health predictions are used to extrapolate all data sensed by the UAS to create a whole-field crop health map. It is assumed that UAS have access to edge computing systems powerful enough for RL and CNN inference. Edge servers or laptops sufficiently augment compute available on UAS and wireless networks allow data transfer between UAS and compute devices. The trade offs between the power and latency of these devices are important to understand, but this is outside the scope of this paper.

#### 2.2.1. Convolutional Neural Network to Model Crop Health

Based on the first law of geography, things that are closer together tend to be more related than things that are far apart, and this is often evident while monitoring agricultural fields [16,29]. For instance, the root causes of poor crop health, such as diseases and pests, often spread to nearby crops. We leverage this property to extrapolate crop health given nearby ground truth. This can be accomplished by providing surrounding zones as the input to a CNN that predicts crop health for a targeted zone.

We chose to modify the VGG16 [28] neural network as our CNN model to predict crop health conditions. Our design trained multiple CNN models, one for each of the eight neighbors adjacent to the center management zone in a 3 × 3 grid. Crop health is computed directly in zones visited by the UAS by using vegetation indices [16]. Given the location of a nearby observation, the corresponding one of these CNN models predicts crop health given the observed image of that zone. These models are designed to leverage the spatial crop health distribution property to predict the condition of the management zones not visited by the UAS. This approach makes a few key assumptions. First, each image captured by UAS represents one management zone. Second, since the UAS captures images in flight, management zones must be connected in the field. This assumption can be problematic in urban settings but reflects common practice in rural environments. In addition, as a corollary, the UAS visit a connected subset of all the management zones in the whole field, which we define as the visible (or observed) area.

**Figure 3:**
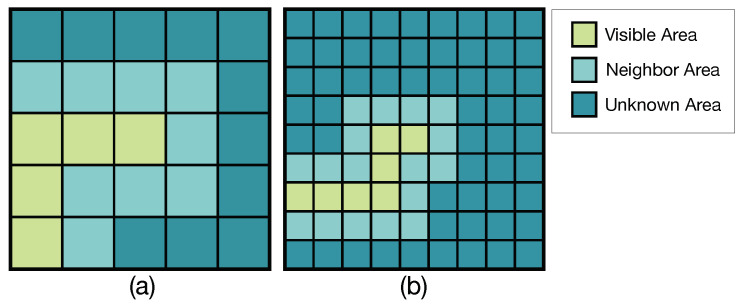
Prediction window examples with size of: (**a**) 5× 5; and (**b**) 9 × 9.

As an illustration, eight observed surrounding management zones represent eight spatial directions regarding the unobserved middle one in a 3 × 3 grid. Eight spatial CNN models were trained by using the surrounding zones of each observation with the label (health condition) of the unobserved center zone as the input. In this case, each of the eight surrounding positions of all zones has a corresponding CNN spatial model that can be used to predict crop health at that direction. Thus, by using CNN, crop health for all the unobserved zones adjacent to the visible area can be predicted. We define these adjacent zones as the neighbor area and the rest zones that are not adjacent to the visible area as the unknown area (Figure 3). Since the neighbor area is adjacent to the flight path (visible area), it is possible for zones in the neighbor area to have multiple adjacent visible zones in their 3 × 3 grid. In this case, predictions from multiple adjacent CNN models are used to improve accuracy, an approach akin to ensemble models.

#### 2.2.2. Crop Health Prediction Algorithm

To generate a real-time crop health prediction map of a field for each flight step of a UAS mission, two questions need to be answered. The first question is how to fulfill an entire crop health prediction map by applying spatial CNN models to the unknown area. To solve this, we introduced the concept of a reference dataset. The reference dataset leverages the fact that the texture of a crop field is similar throughout, which means you can potentially find two zones that are quite similar given enough samples. The reference dataset consists of many zone images that are not used in the training dataset. Instead of the final prediction (1 or 0), each zone image is associated with the prediction with confidence (i.e., 70.45% confidence in healthy condition) from CNN model. The function of the reference dataset is as follows: every time a zone in the neighbor area gets a prediction and confidence value from spatial CNN models, the number is then compared with numbers in the reference dataset. The image having the closest prediction confidence is chosen and placed at the position of the zone. In this way, zones in the neighbor area not only get predicted health conditions, but also gain visible images, which can be treated as visible area later. The second question is how to generate a crop health map of a field quickly such that UAS do not waste power hovering while waiting for movement instructions. For a large crop field, the health condition of a zone has almost no influence on a zone that is 20 zones away. Thus, for each flight step, we only need to consider creating the crop health map within some close distance of the current UAS position. We defined the UAS-centered range as the prediction window.

**Figure 4:**
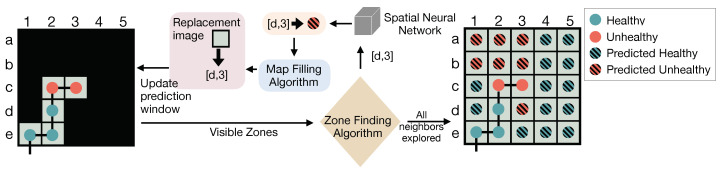
Crop health map prediction algorithm within a prediction window.

Based on the reference dataset and the prediction window, we developed a two-stage crop health prediction algorithm to extrapolate crop health information beyond the neighbor area to reach out to the unknown area and create instant crop health maps. First, as shown in Figure 4, the algorithm initiates a prediction window with a chosen size. The initial window only contains the sensed data of all zones on the flight path (visible area). Next, spatial CNN models are used to predict the health conditions of zones in the neighbor area. After the prediction for a given zone is obtained, the crop health map is updated by adding the health condition and a replacement image from the reference dataset at the position of the zone to expand the visible area. Then, the neighbor area and the unknown area will be updated as well. This process continues until the prediction window has no unknown area. In this way, the algorithm keeps filling the prediction window with zone image, making it possible to predict all zones that are not originally adjacent to the visible area.

#### 2.2.3. Reinforcement Learning Algorithm

Once crop health conditions are evaluated, we use a RL algorithm from prior work [17], a modified version of Q-learning, to determine the next UAS movement direction. UAS are designed to maintain one map for every flight step while exploring a crop field. Each map is one state of the field that uses two kinds of information, collected crop health conditions (aerial images on the flight path) as the ground truth data and predicted crop health generated by the crop health prediction algorithm. These data are used to develop a state model which provides a series of possible flight directions from the current zone. The final flight decision is made by the state model using a list of prior observations of similar field states. To build a dataset with plenty of field states, we developed an algorithm to simulate a random flight path and collect map data (detailed in Section 2.4.3).

The model was trained with a dataset of 73,000 unique field state combinations. The k-nearest neighbors (KNN) [30] algorithm was used to determine which prior examples are most relevant for a given combination of ground truth and prediction maps. KNN determines the 11 most similar prior states to the current state. To determine the final movement direction, RL compares predicted labels for each flight action from KNN to CNN predictions. The flight action with the largest distance between its KNN and CNN prediction (i.e., highest error) is chosen to ensure that UAS explore the locations that they least understand.

### 2.3. Local-Field RL Algorithm

Local-Field RL is similar in many ways to whole-field RL. Both algorithms use RL to navigate the field and generate a final crop health map. There are, however, a series of important differences between them. Unlike whole-field RL which uses a prediction map generated from the crop health prediction algorithm as an input to RL, local-field RL extracts image properties (i.e., ExG, RGB saturation) from visible zones and feeds those data as an input to RL. Once the UAS has covered a certain amount of the field, local-field RL extrapolates the crop health map of a field using a KNN-based recursive dilation procedure instead of relying on a CNN. The dilation procedure finds every management zone in the map that has not been predicted or observed and assigns that zone the consensus of its directly adjacent neighbors, if it has any. If it has no neighbors, the position remains unassigned. This process is performed recursively until the entire map is full.

### 2.4. Implementing Autonomous Aerial Scouting

Autonomous scouting algorithms were implemented using the SoftwarePilot simulation environment [31], which was used in prior work to implement and simulate local-field RL [11,17]. In this study, SoftwarePilot was modified to use CNN-based model outputs for crop health map prediction as well as for RL-based path finding algorithms. This simulator performs both the local-field and whole-field RL algorithms until a user-specified coverage of the crop field is explored, then uses the extrapolation algorithms to predict any areas of the final map that are still empty.

#### 2.4.1. Dataset

CNN training and flight simulation were performed on a dataset collected from a corn field at the Molly Caren Research farm near London, Ohio in August 2017. This dataset includes 684 aerial images with a resolution of 4608 × 3456 pixels in RGB channels. Images were collected at 200 feet from the ground using an eBee UAS from senseFly with a ground sample distance of 1.9 cm/px. For this study, we used 30 aerial images as our training dataset, 6 aerial images as the test set and another 14 images as the reference dataset. Each image is broken into a set of 1344 zones in a 42 × 32 grid. Each zone is around 4.3 square meters.

#### 2.4.2. Whole-Field RL Implementation

CNN-based crop health modeling is used in two parts of the experiment. First, we need to use the crop health prediction models to build datasets for the RL algorithms. Second, during simulation, the crop health prediction models provide near real-time predicted crop health map for each management zone the UAS captures and for the whole field based on the final flight path. In this subsection we mainly discuss RL dataset construction. The extrapolation procedure is discussed in the following subsection.

To build a large RL dataset and perform thorough analysis, datasets were prepared using six coverage rates: 10%, 20%, 30%, 40%, 50% and 60%. For each coverage rate, five whole-field RL prediction window sizes—7 × 7, 11 × 11, 15 × 15, 19 × 19 and 23 × 23—were used. For each combination, 1000 flight paths were randomly generated. The process begins with choosing a random start point for the UAS on the edge of the field. Each subsequent flight step is chosen randomly from the neighboring management zones of the current position. The simulated flight path keeps growing until it meets the specified coverage rate. If the UAS has sampled all its directly adjacent neighbors, but has not reached the coverage threshold, it flies to the nearest unsampled management zone. For each zone the UAS visited, the crop health map for the prediction window is calculated using the crop health prediction algorithm. Crop health is estimated based on ExG [16], a vegetation index derived using pixel values from aerial photographs. These data are also used as ground truth. Average ExG of each zone is compared with the average ExG of the entire field. If the zone ExG is less than 80% of the average field ExG, then it is classified as unhealthy, and, if the ExG is at least 80% of the average ExG, the zone is labeled as healthy.

#### 2.4.3. Simulation Environment

Our simulation environment was modified from SoftwarePilot [31], the local-field RL simulator. For the purpose of simulation, we considered each image in the test set to represent the flight area of the fully autonomous aerial scouting system, and for each zone to represent sensed data from the simulated UAS. The experiment was conducted with a Lenovo ThinkPad T470 as the edge system. This system has an i7-7500u processor, 24GB of RAM and runs Ubuntu 18.04.

### 2.5. Non-Autonomous Scouting Approach

Other than the traditional exhaustive scouting approach and local-field RL, we also compared our whole-field RL algorithm with two naive approaches: random scouting and non-scouting. Random scouting entails UAS randomly choosing flight directions until the provided coverage rate is reached. Non-scouting refers to the naive approach of applying fertilizers uniformly without considering internal field variability.

### 2.6. Comparison between Scouting Approach

We compared our whole-field RL scouting approach with the local-field RL approach and some traditional methods (the exhaustive, random scouting and non-scouting approaches) based on metrics such as accuracy, positive precision, positive recall, negative precision and negative recall (discussed below) relative to the ground truth health determined using ExG. Positive and negative indicate healthy and unhealthy crop conditions, respectively. Positive recall represents the ratio between all correctly classified true positives and all true positives (both true positives and false negatives), where a higher ratio represents efficient avoidance of false negatives. Negative precision similarly represents the ratio of correctly classified true negatives to all true negatives (classified true negatives and classified false positives), where a high negative precision represents an efficient avoidance of false positives. While false positives refer to unhealthy management zones that are misclassified as healthy, false negatives refer to healthy management zones that are misclassified as unhealthy. True positives and negatives indicate management zones that are correctly classified as healthy and unhealthy. Management decisions based on false positives can result in untreated crops, which may result in low crop yield. Similarly, if unhealthy management zones are predicted as healthy (i.e., false negative), they could lead to excessive use of resources such as fertilizer thereby increasing the likelihood of higher nutrient load to air or water.

#### 2.6.1. Energy and Labor Costs Estimation

To compare the performance of all approaches from energy and cost perspectives, a simple cost–benefit model was developed by adding up revenue based on crop yield from all the management zones and subtracting the cost of treating misclassified zones (healthy classified as unhealthy and vice versa) as well as UAS deployment costs. The labor costs were considered to be $10 and $20/h for unskilled and skilled workers, respectively [32]. It was also assumed that autonomous scouting approaches require only one unskilled worker to complete the entire survey, whereas exhaustive scouting requires an additional skilled worker (i.e., two in total) to plan and complete UAS surveys including setting up the system, planning the routes and swapping UAS batteries. In the non-scouting approach, it was assumed that farmers classify every zone as unhealthy and thus treat the field equally.

#### 2.6.2. Nutrient Runoff Risk

We estimated potential risk of nutrient runoffs under various scouting approaches with two assumptions: (1) farmers tend to apply fertilizer uniformly throughout a field if they do not have site specific information from scouting (i.e., non-scouting), and thus the nutrient runoff risk of a field is 100%; and (2) if they have site specific information (i.e., various types of scouting), they apply treatments only to poor (i.e., unhealthy) sections of a field, which reduces the nutrient runoff risk. Thus, the potential of autonomous scouting approaches to reduce nutrient runoff risks is dependent directly on the false negative rates from the classification as unnecessary nutrient runoffs can occur when healthy zones are unnecessarily fertilized. To determine how these two autonomous scouting approaches help minimize nutrient runoff risk, we estimated the percentage of healthy zones that are unnecessarily fertilized.

## 3. Results and Discussion

In this study, the accuracy, scouting cost, revenue and energy consumption of the proposed fully autonomous scouting techniques were assessed and compared with state of the practice automated scouting and non-scouting approaches used in both precision agriculture and general agriculture.

### 3.1. Comparing Fully Autonomous Aerial Scouting and Conventional Methods

**Figure 5:**
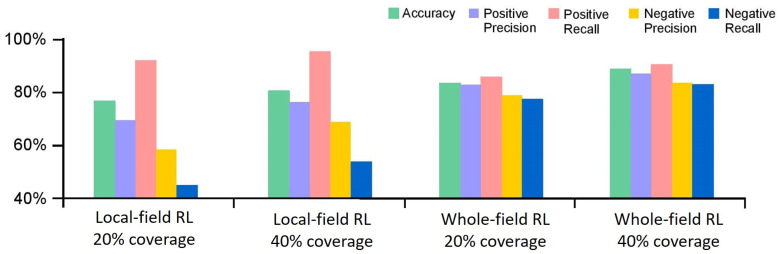
Accuracy of maps generated by autonomous scouting at different coverage rates.

Accuracy differences between whole-field and local-field RL at both 20% and 40% coverage settings were compared in Figure 5, which showed that whole-field RL at 20% coverage provides 2.3% better accuracy than local-field RL at 40% coverage. Local-field RL provided 74.5% and 80.3% accuracy at 20% and 40% coverage, respectively. This is compared to 82.6% and 87.3% accuracy at 20% and 40% coverage, respectively, for whole-field RL.

Local-field RL outperformed whole-field RL considerably at avoiding false negatives. It experienced 8.3% and 8% higher positive recall than that of whole-field RL at 20% and 40% coverage, respectively. However, there was a 67% increase in negative recall for whole-field RL over local-field RL at 20% coverage, and 58% at 40% coverage.

As shown in Table 2, when comparing the two autonomous scouting methods for coverage rates of 10–60%, whole-field RL outperformed local-field RL considerably between 20% and 50% coverage while local-field RL outperformed whole-field RL at 10% coverage with a higher positive precision rate and fewer false positives. At 10% coverage, local-field RL classified nearly the entire field as healthy compared to whole-field RL. At 60% coverage, there was a small difference in overall accuracy between whole-field and local-field RL; however, negative recall was significantly higher in whole-field RL. While local-field RL experienced consistent accuracy gains as coverage improved, whole-field RL experienced gains at lower coverage, with a considerable drop-off at 50%; its performance is restricted by our CNNs, whose average accuracy is around 90%. This suggests that the marginal benefit of increasing whole-field RL coverage past 60% is likely not worth the marginal cost of labor and equipment. Over 60% coverage, the extra cost is more than the extra revenue compared to a 40% coverage rate.

The main difference between the two autonomous scouting approaches is how they consider surrounding zones for prediction of crop health and pathfinding. Whole-field RL uses a CNN-based prediction window for path finding, in which a few poor management zones can reinforce the scouting of poor management zones in their surroundings. Local-field RL simply uses a KNN-based linear approach, which predicts health condition of a management zone based on its adjacent neighbors, and thus can achieve high positive recall. While it is important to achieve high positive recall, negative recall can have significant cost implications when implementing site-specific management practices. Treating an unhealthy zone as healthy (negative recall) is estimated to cost eight times higher than treating a healthy zone as unhealthy (positive recall) as discussed in Section 2.6.

### 3.2. Autonomous Pathfinding and Extrapolation Comparison

**Figure 6:**
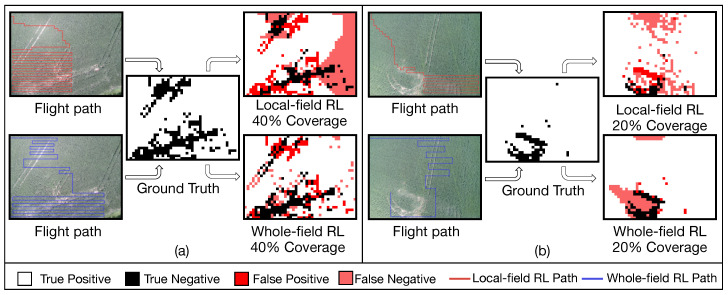
Different crop health maps and flight paths generated by whole-field and local-field RL at 20% and 40% coverage.

Both autonomous scouting approaches appear to alternate between two natural behaviors, exploration and scouting. Exploration involves the UAS traversing the field, covering large swaths in search of a region that contradicts current map conditions. Scouting involves the UAS moving in an exhaustive fashion across a region that the RL algorithm perceives as important. Whole-field RL was found to take better advantage of these two pathfinding behaviors by quickly finding areas that it perceives to be problematic, and more thoroughly scouting those areas. This contrasts with the local-field RL approach where the first cluster in Figure 6a is traversed but barely explored and a large chunk of the second cluster is ignored. These discrepancies are likely due to the quality of the inputs provided to the RL algorithm in each approach. Whole-field RL provides its entire prediction and ground truth windows which more accurately locate relevant prior examples in the dataset than the local features used in local-field RL.

However, as illustrated in Figure 6b, local-field RL outperformed whole-field RL in some cases at a low coverage rate. Distracted by traces of tractors, whole-field RL spends a considerable amount of time scouting a questionable narrow area in the top left of the field, while local-field RL rushes to the bottom to explore a region that is partially unhealthy. While whole-field RL eventually finds the bounds of the large negative cluster, the quick decision by local-field RL that leads to finding the bad cluster earlier leads to improved accuracy at this low coverage setting.

Differences between the local-field and whole-field RL extrapolation methods are also apparent when examining the four output maps. Both local-field RL maps show significant clusters of false negatives (pink areas). This is due to the underlying KNN-based extrapolation algorithm. When local-field RL’s extrapolation algorithm encounters a cluster of similarly classified points, it tends to reinforce that classification across nearby unpredicted zones. One cluster of negative zones would be easily extrapolated such that a huge area of a field could be falsely predicted as negative, which is shown in predicted crop health map by local-field RL in Figure 6a. This behavior is not as apparent in whole-field RL which uses online predictions to fill zones instead of binary extrapolation.

### 3.3. Autonomous Scouting on Fields with Various Crop Health Conditions

**Figure 7:**
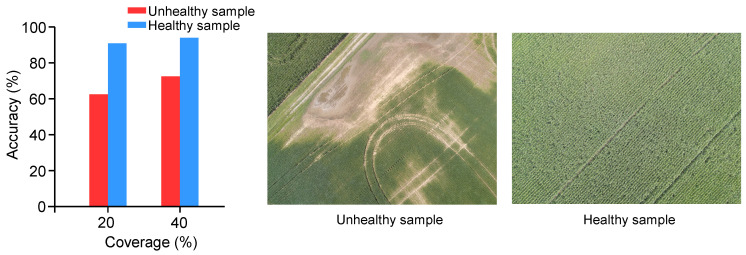
Accuracy of whole-field RL at 20% and 40% coverage rates for largely healthy and largely unhealthy samples.

The performance of whole-field RL was explored on two very different regions, one which is primarily comprised of healthy zones and the other primarily comprised of unhealthy zones (Figure 7). In the first image, crop density was lower in portions of the field where areas appeared to be compacted by agricultural machinery. Other portions appeared to be low elevation areas, where crops emergence was impacted by prolonged saturation of water (i.e., ponding). In contrast, the second image showed a healthy corn field where lush green corn rows can be seen, separated only slightly by intermittent gaps.

From the chart in Figure 7, we can see that whole-field RL achieved over 90% accuracy for both coverage rates in the healthy sample. The accuracy increased over the average accuracy of the total dataset is due largely to the uniformity of the healthy image, which indicates that whole-field RL can accurately extrapolate from a set of entirely healthy management zones. However, the size of the reference set impacts accuracy. Sometimes unknown healthy images can be replaced with unhealthy images due to their similarity in comparison to the rest of the reference set, which keeps whole-field RL from achieving 100% accuracy on sample healthy image at both coverage rates. Doubling coverage only improved accuracy by 3.2% for the healthy image. In contrast, doubling coverage for the unhealthy image improved accuracy by 10.3%.

The uniformity of the healthy image limits the accuracy gains from increasing coverage, which simply decreases the prevalence of false negatives generated erroneously by the reference set. Increased coverage in the non-uniform unhealthy image allows the system to get a better understanding of the topology of negative clusters to improve extrapolation. The importance of high coverage is apparent for predominantly unhealthy fields, but the low net accuracy must also be explained. Whole-field RL is largely negative-biased as discussed previously. When confronted with a majority negative image, the extrapolation procedure will reinforce negative regions, resulting in a larger number of false negatives than we see in, for instance, the healthy image in Figure 7. While whole-field RL experiences decreased overall accuracy on predominantly negative fields due to a high false negative rate, the decreased cost of treating false negatives as compared to false positives discussed earlier in this section implies only modest losses from treatment costs as compared to crop loss from such incorrectly predicted zones.

### 3.4. Effects of Prediction Window Size

**Figure 8:**
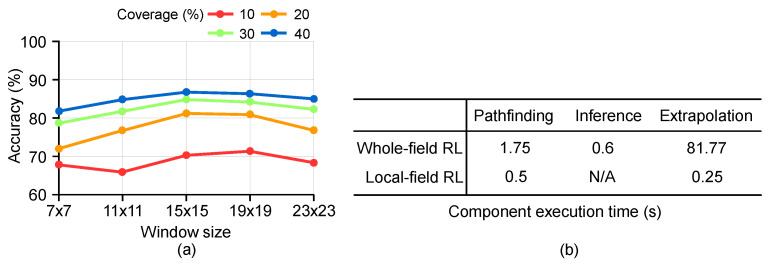
(**a**) The effects of prediction window size on final crop health map accuracy at different coverage rates for whole-field RL; and (**b**) the execution times of software components for local-field RL and whole-field RL with a window size of 15. [Note: Crop health map was generated offline.]

While comparing different combinations of prediction window sizes and coverage rates on accuracy, it was found that increasing prediction window size is not always beneficial. As shown in Figure 8a, for 20–40% coverage rate, accuracy is highest for the 15 × 15 window size, with lower accuracy proportional to both increases and decreases of the window size. Ten percent coverage rate had the highest accuracy at a window size of 19. Accuracy decreases at smaller window sizes can be attributed to a lack of iteratively updated prediction information with which to generate a final map. As the UAS moves around the field, it iteratively updates unseen but nearby areas to the flight path. If this window does not extend out far enough from the flight path, the only update that some areas will receive is the final extrapolation. At smaller window sizes, it is clear that some areas could have benefited from iterative updates, which would in turn increase accuracy. The opposite can be said for accuracy decreases with increased window size. If the prediction window is too large, the CNN approach may not be able to accurately predict their health due to their distance from ground truth. This result is critical to performance. Given the quadratic increase in latency as the prediction window increases, it is imperative that a whole-field RL system balances accuracy against increased costs due to latency. Given that we have found accuracy’s inflection point as a function of window size, a simple solution could be to use the most accurate window size, which we have done for these experiments.

It is worth noting that, as prediction window size increases, so does the size of the RL dataset required for pathfinding. Both the increased number of predictions and larger pathfinding overhead increase system latency, so larger windows should be avoided to increase throughput unless accuracy returns justify them. From Figure 8b we can see that the process to compute the final crop health map at the edge system offline by extrapolation after a mission is complete took on average 320X longer for whole-field RL than for local-field RL. Whole-field RL’s CNN models required considerably more time due to computational complexities than local-field RL’s KNN-based approach. This process is, however, performed offline. Latencies simply determine how long the farmer must wait after mission execution to receive a crop health map. While whole-field RL experiences much higher latency in crop health map generation, both approaches return a map to the farmer in reasonable time.

### 3.5. Energy, Labor Costs and Nutrient Runoff Risk

**Figure 9:**
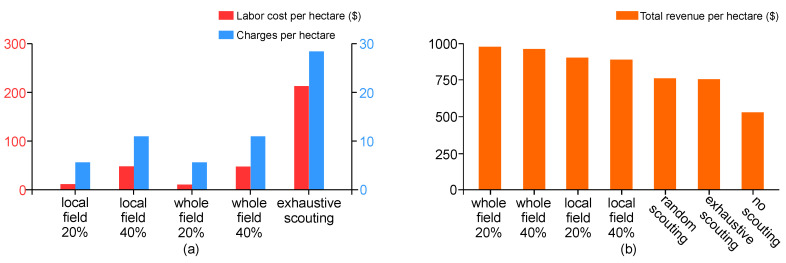
(**a**) Energy implementations and labor costs of autonomous scouting vs. exhaustive scouting; and (**b**) the impacts that autonomous scouting have on revenue compared to state of the practice methods.

As shown in Figure 9a, the amount of charges required to map a hectare of crops differs between three scouting approaches due to the percent of field area covered in each approach. Since it was assumed that the same number of charges would be required to cover the same size of a field across all scouting approaches, local-field and whole-field RL experienced the same charges at the same coverage setting, requiring 6 charges at 20% coverage and 12 charges at 40% coverage. This compares to 29 charges to map one hectare of land using the traditional exhaustive scouting method which was found to require considerably more labor and charges to complete the scouting mission.

There were significant differences in labor costs for different scouting approaches compared in Figure 9a. Considering the economic data from the 2018 growing season [32], at hourly rates of $10 and $20 for unskilled and skilled workers, respectively, autonomous scouting methods were roughly estimated to cost $29 and $44 for 20% and 40% coverage of an 80-acre crop field, which compares to the $212 mapping cost for exhaustive mapping using two laborers.

According to recently published agricultural cost data [32], the revenue per acre for corn is $763.8 USD ($3.8/bushel × 200 bushels/acre). Revenue per management zone is calculated to be $0.8 for the size of 4.3 square meters per zone. The cost of fertilizer per acre is $130, which makes it $0.1 per management zone. Thus, one false negative management zone would cost a farmer $0.8 due to crop loss, while one false positive management zone would cost $0.1 in treatment cost. Based on this, when the field is not scouted, it is estimated to provide 36% less revenue than whole-field RL, and 27% less revenue than local-field RL as shown in Figure 9b. Exhaustive scouting outperformed no scouting by 20% but loses out to local-field and whole-field RL by 5% and 17%, respectively. While exhaustive scouting will provide 100% accuracy, allowing farmers to properly treat their entire field, the labor costs of exhaustively mapping large fields is outweighed by lower coverage autonomous mapping with extrapolation. We also explored the effects of a random sampling approach at 40% coverage using local-field RL. This automated approach without RL pathfinding underperforms compared to exhaustive scouting by 1.2%, demonstrating that not all automated and naïve autonomous approaches are superior.

Between autonomous approaches at 40% coverage, we found that whole-field RL garnered 13% more revenue than local-field RL. Despite similar labor costs, the accuracy improvements over local-field RL, particularly among the negative recall, provides a considerable increase in revenue for whole-field RL over local-field RL. By limiting false negatives, whole-field RL was found to reduce higher runoff risk by 12% compared to local-field RL. However, all the revenue data were generated from our simulation environment, which means that they represent the best case scenarios without considering other important factors such as climate, weather, market and insects.

## 4. Limitations and Future Work

Autonomous scouting methods inherently avoid surveying 100% of a field to save time, energy and money. This, however, runs the risk of missing critical field health problems. This problem can be minimized if a field is regularly monitored for potential crop health problems during growing seasons. A problematic section of a field that might not have been picked up by autonomous scouting at one time is likely to be picked up if the field is regularly mapped.

The cost–benefit model used in this study considers fertilizer application as the only treatment for unhealthy zones. Zones may have stresses such as pest and water other than nutrients only, and thus need to be treated accordingly, which could in turn influence the treatment costs. In addition, costs and benefits were estimated based on corn only. Some of these estimates can differ by crop and treatment types. Future studies should be focused on evaluating some of these factors.

In the study, we used ExG as an indicator of crop health for simplicity. There are however other vegetation indices (e.g., NDVI and green index) and biophysical variables (e.g., soil organic carbon, pH and elevation) that are also reported to be good indicators of crop health [33]. Future studies could exploit a combination of these variables as indicators of crop health while developing models for autonomous scouting approaches.

For crop health prediction, we used an ensemble of spatial CNN models. Other deep learning methods, such as semantic segmentation, should also be considered. Semantic segmentation classifies individual pixels in an image, providing a more detailed view of crop health. Yang et al. (2020) used semantic segmentation in the detection of rice lodging [24]. Long et al. designed fully convolutional networks for semantic segmentation which could be adapted in our CNN model for crop health prediction [34]. Besides, considering the improper proportion of healthy and unhealthy zones, Yu and Fan designed a deep semantic labeling framework with special consideration of rare classes that could be used for detecting sparse unhealthy zones [35].

Future work should also focus on training, reinforcement learning and testing of the models based on data collected from with variety of crops under different field conditions. The RL approaches used in this study, which are similar to q-learning, can also be compared with other related sampling algorithms such as rapidly-exploring random trees. Similarly, future work should address how prediction window size and its effect on architectural latency affects accuracy and overall cost of the system.

## 5. Conclusions

In this paper, we design and discuss a new fully autonomous aerial scouting approach, whole-field RL, which we compare to local-field RL and the current naive UAS approach of exhaustive scouting. The performance of these two RL approaches along with other popular scouting methods was assessed in terms of accuracy, precision, recall, execution time of crop health map generation and cost-saving potential across different field coverage rates. Compared to local-field RL, whole-field RL can boost accuracy of crop health maps by 9%. This approach produces accurate crop health maps after flying over only 40% of the field. Whole-field RL reduced labor cost by 4.8 times compared to naive methods, increased agricultural profits by 36% and reduced runoff potential by 87%. We found that coverage rate offers diminishing improvements in accuracy after 40%. The considerable improvement in performance of whole-field scouting over local-field scouting can largely be attributed to its added CNN models, which use surrounding ground truth data to predict the health conditions of management zones in flight. In-flight predictions allow the final crop health map to be iteratively updated and the flight path to be refined online, producing a more accurate final product. Our evaluation shows that fully autonomous aerial scouting can guide crop field management techniques using less money and less agricultural product while garnering greater monetary returns than the state of the practice.

## Figures and Tables

**Table 1:** The benefits of the fully autonomous aerial scouting captured by empirical traces of battery drain.

Method	Exhaustive Scouting								Fully Autonomous Aerial Scounting				
Mission%	0	4	20	36	52	68	84	100	0	10	50	90	100
Step	0	1	5	9	13	17	21	25	0	1	5	9	10
Battery%	100	95	75	55	35	85	65	45	100	95	75	55	50
Current Zone		[e,0]	[a,0]	[d,1]	[c,2]	[b,3]	[e,4]	[a,4]		[e,0]	[d,3]	[c,4]	[d,4]

**Table 2:** Accuracy, precision and recall for maps generated using whole-field and local-field RL at different coverage rates.

Coverage Rate		10%	20%	30%	40%	50%	60%
Local Field RL	Accuracy	0.73	0.75	0.77	0.80	0.84	0.88
	Positive Precision	0.69	0.69	0.72	0.75	0.79	0.83
	Positive Recall	0.89	0.91	0.93	0.94	0.95	0.97
	Negative Precision	0.49	0.57	0.64	0.72	0.78	0.85
	Negative Recall	0.48	0.46	0.46	0.51	0.59	0.61
Whole Field RL	Accuracy	0.70	0.83	0.85	0.87	0.89	0.90
	Positive Precision	0.71	0.83	0.85	0.87	0.89	0.89
	Positive Recall	0.71	0.84	0.87	0.89	0.93	0.94
	Negative Precision	0.64	0.78	0.81	0.82	0.85	0.87
	Negative Recall	0.65	0.77	0.80	0.81	0.83	0.83
